# Supplementary material for: Relationship of taxonomic error to frequency of observation
Source: PLoS One. 2020 Nov 12;15(11):e0241933. doi: 10.1371/journal.pone.0241933 (PMC7660486; doi:10.1371/journal.pone.0241933)
Supplement: Attachment — Submitted filename: Response to reviewers.docx [file pone.0241933.s003.docx]

**Stribling and Leppo, 10/09/20**

**“*Relationship of taxonomic error to frequency of observation*” PONE-D-20-27394**

**Responses to editor and reviewer comments and suggestions**

Author responses **(IN RED)**

**To the Editor:**

1. Please ensure that your manuscript meets PLOS ONE's style requirements, including those for file naming. The PLOS ONE style templates can be found at

https://journals.plos.org/plosone/s/file?id=wjVg/PLOSOne_formatting_sample_main_body.pdf and

https://journals.plos.org/plosone/s/file?id=ba62/PLOSOne_formatting_sample_title_authors_affiliations.pdf

DONE

2. Thank you for stating the following in the Financial Disclosure section:

"Approximately 10% of necessary level of effort in initiating this project was contracted to Tetra Tech, Inc. (JBS) (EP-C-14-016, Work Assignment 4-13) by the US Environmental Protection Agency/Office of Water/Office of Wetlands, Oceans, and Watersheds/Assessment and Watershed Protection Division. The work was in support of the Agency's National Aquatic Resources Surveys:

https://www.epa.gov/national-aquatic-resource-surveys

The sponsors played no role in the study design, data collection and analysis, decision to publish, or preparation of the manuscript."

We note that you received funding from a commercial source: Tetra Tech, Inc.

* Please provide an amended Competing Interests Statement that explicitly states this commercial funder, along with any other relevant declarations relating to employment, consultancy, patents, products in development, marketed products, etc.

* Within this Competing Interests Statement, please confirm that this does not alter your adherence to all PLOS ONE policies on sharing data and materials by including the following statement: "This does not alter our adherence to PLOS ONE policies on sharing data and materials.” (as detailed online in our guide for authors http://journals.plos.org/plosone/s/competing-interests). If there are restrictions on sharing of data and/or materials, please state these. Please note that we cannot proceed with consideration of your article until this information has been declared.

* Please include your amended Competing Interests Statement within your cover letter. We will change the online submission form on your behalf.

Our “Revised Financial Disclosure Statement” follows and is included in the cover letter.

Approximately 10% of necessary level of effort in initiating this project was contracted to Tetra Tech, Inc. (JBS) (EP-C-14-016, Work Assignment 4-13) by the US Environmental Protection Agency/Office of Water/Office of Wetlands, Oceans, and Watersheds/Assessment and Watershed Protection Division. The work was in support of the Agency's National Aquatic Resources Surveys:

<https://www.epa.gov/national-aquatic-resource-surveys>

Additionally, Tetra Tech, Inc., the employer of JBS, allowed company resources to be applied to some of the data analyses and manuscript preparation, in particular, computer hardware, software and network resources, and limited labor hours.

The sponsors played no role in the study design, data collection and analysis, decision to publish, or preparation of the manuscript. They also have no interests relating to employment, consultancy, patents, products in development, marketed products, etc. This revision does not alter our adherence to PLOS ONE policies on sharing data and materials.

Please know it is PLOS ONE policy for corresponding authors to declare, on behalf of all authors, all potential competing interests for the purposes of transparency. PLOS defines a competing interest as anything that interferes with, or could reasonably be perceived as interfering with, the full and objective presentation, peer review, editorial decision-making, or publication of research or non-research articles submitted to one of the journals. Competing interests can be financial or non-financial, professional, or personal. Competing interests can arise in relationship to an organization or another person. Please follow this link to our website for more details on competing interests: http://journals.plos.org/plosone/s/competing-interests

3.

Please amend the manuscript submission data (via Edit Submission) to include author:

Erik W. Leppo

YES

**To Reviewer #1**

1. Lines 221, 226, 229, 230, 233-4, 239, 242 can you provide context to the taxonomic groups (e.g., order) the mentioned taxa belong, as done for Nais (line 222)? For a non-taxonomist or ecologist without knowledge of North America macroinvertebrates this would help immensely rather than the having to cross reference with supplementary files. I APPRECIATE THIS COMMENT. HOWEVER, IS IT NOT SUFFICIENT THAT THE TAXA MENTIONED IN THIS SECTION, AS REPRESENTATIVE AND SELECTED EXAMPLES, ARE GIVEN WITH THEIR CLASSIFICATION HIERARCY IN TABLE 4? I WOULD CONTEND THAT IT ***IS*** SUFFICIENT, AND ACTUALLY MAKES THE TEXT AN EASIER READ WITHOUT THE INLINE PARENTHETICALS. FOR CONSISTENCY, I DELETED THE HIERARCHY PARENTHETICAL FOR *NAIS*.

2. Although there is no mention to macroinvertebrate size (in relation to instar size) can you comment if instar size correlates to an increase in RPD error? THE STRIBLING ET AL. 2008 PAPER CITED GOES INTO SOME OF THE CAUSES AND MAGNITUDES OF ERROR. BUT YES, BODY SIZE, LIFE STAGE, AND SPECIMEN CONDITION ALL PLAY AN OUTSIZED ROLE IN IDENTIFICATION ERROR. THE PART OF THAT WHICH IS INTERESTING IS THAT TAXONOMISTS THAT ARE MORE EXPERIENCED AND WITH MORE IN-DEPTH TRAINING ARE ABLE TO OBTAIN SOLID RESULTS (=ACCURATE, CONSISTENT) MORE EASILY THAN THOSE WHO ARE LESS EXPERIENCED. I ADDED THE FOLLOWING TEXT IN THE DISCUSSION SECTION: “Another potential use of these results would be in helping target individual taxa for determining causes, beyond lack of familiarity, of higher error rates. A common cause is known to be specimens in poor condition and/or small body size (early life stages, or instars). An outcome of such an investigation might be to specify standard procedures for some taxa, including for sampling, handling, preservation, and identification. An example of this might be a requirement that all larval Chironomidae be slide-mounted for examination under a compound microscope. We do not necessarily advocate this, as slide-mounting is not consistently needed by all laboratories or taxonomists. Rather, we stress that the taxonomist use whatever method is needed to attain the target taxonomic level as defined by program or study goals. The goal in this case is not to require that all taxonomists (or taxonomic technicians) slide-mount all chironomid midges; rather, the goal is to acquire genus level data for the taxon. In some cases, slide-mounting might be needed, in others, it would not. Thus, the need for such actions would be determined on a case-by-case, taxon-by-taxon, or even taxonomist-by-taxonomist basis, but the goal of genus level data remains the same.”

3. Lines 222, 224, 232. Consistency required for expressing percentages. I AM UNSURE OF THE ISSUE HERE, AND WHAT CHANGES ARE BEING REQUESTED.

4. Figure 1. Can this axis extend to 100% as seen in Figure 5? DONE Do this also for Fig. 3. DONE The titles of X and Y axes are in upper case and not consistent with the other figures. CORRECTED

5. Figures 3, 4 and 5. There is considerably overlap in these three figures. Figure 3 is not necessary as this information is repeated in Figure 5. Although there is subtle difference in how these data points are plotted, they are essentially the same graph. Instead, can figures 4 and 5 be combined? For example, can each of the data points that correspond to a UFC categories be colour coded or superimposed on Figure 4? I BELIEVE ALL THREE FIGURES ARE NECESSARY FOR THE PAPER. FIGURE 3 PROVIDES THE READER WITH AN UNCOMPLICATED VISUALIZATION OF THE DATA DISTRIBUTION, FIGURE 4 IS A DEMONSTRATION OF THE CATEGORIES, AND FIGURE 5 ACCOMPLISHES THE SUGGESTED SUPERIMPOSITION. IT JUST SEEMS LIKE THERE IS BETTER CLARITY IN HOW THE UFC STRUCTURE IS PRESENTED IF ALL THREE ARE RETAINED. AS THAT IS THE CORE FOUNDATION OF THIS ANALYSIS, I BELIEVE IT IS BEST PRESENTED AS IT IS.

6. Figure 6. A 2D rather than a 3D plot would be preferred. A legend is required (1 = High confidence, common; 2 = High confidence, moderately common etc) to avoid the reader having to reference back to Figure 4. CORRECTION MADE; LEGEND ADDED TO CAPTION.

7. Figure 7. This figure needs to be produced in a similar format to Figure 1, i.e., consistent formatting of Y and X axis titles. As noted above, it is suggested a legend is added to this figure. I WILL ADD THE LEGEND TO THE CAPTION. HOWEVER, I THINK I DISAGREE WITH THIS SUGGESTION ABOUT THE BARCHART STYLE. THE TWO CHARTS ARE SHOWING DIFFERERNT KINDS OF DATA, AND BY USING STACKED BAR CHARTS/PERCENTAGES, MUCH SPACE IS SAVED. THAT IS, I FEEL CONFIDENT THIS IS THE MOST EFFICIENT TECHNIQUE FOR SHOWING HOW EACH OF THE MAJOR TAXA SORT AMONG THE CATEGORIES.

8. Figure 8. Y axis uses “FREQ(%)” but “FREQ” is used elsewhere. THESE FIGURE PANELS ARE UNIQUE, AND I BELIEVE THEY WARRANT SOMEWHAT UNIQUE AXIS TITLES. I WOULD LIKE TO OPT TOWARD SPELLING THEM OUT, SUCH AS, FROM TOP TO BOTTOM: “Frequency of observation (FREQ)” and “Relative percent difference (RPD)”. Note the use of “/” in manuscript text but hyphen in X axis and figure caption (line 207). See also lines 204, 207 and replace hyphen with “/”. DONE. This figure also requires a legend for the frequency classes. I ADDED A LEGEND TO THE CAPTION FOR THIS, AS WITH THE PRIOR FIGURES.

9. In the discussion are you able to provide an overview or summary of which groups you found to be more problematic than others and, for example, are likely in need for more comprehensive or updated identification guides. THE FOLLOWING TEXT WAS ADDED TO THE DISCUSSION SECTION: “Higher level macroinvertebrate taxa in this analysis shown to have greater identification confidence and consistency are midges (Insecta: Diptera: Chironomidae), caddisflies (Insecta: Trichoptera), beetles (Insecta: Coleoptera), snails (Mollusca: Gastropoda), and stoneflies (Insecta: Plecoptera) (Fig 7), as they are mostly made up of finer level taxa with UFC1-3. Conversely, higher level taxa for which identification data seem to be more problematic (i.e., greater uncertainty) are bivalves (Mollusca: Pelecypoda) and Crustacea (Arthropoda). Each of these groups have a higher percentage of taxa in UFC4-5.”
